# Intranasal Single-Replication Influenza Vector Induces Cross-Reactive Serum and Mucosal Antibodies against SARS-CoV-2 Variants

**DOI:** 10.3390/vaccines11061063

**Published:** 2023-06-05

**Authors:** Michael J. Moser, Lindsay Hill-Batorski, Richard A. Bowen, Sarah M. Matejka, David Marshall, Yoshihiro Kawaoka, Gabriele Neumann, Pamuk Bilsel

**Affiliations:** 1 FluGen, Inc., 597 Science Drive, Madison, WI 53711, USA; 2 Department of Biomedical Sciences, Colorado State University, 1601 Campus Delivery, Fort Collins, CO 80523, USA; 3 Department of Pathobiological Sciences, University of Wisconsin, 2015 Linden Dr., Madison, WI 53706, USAgabrielle.neumann@wisc.edu (G.N.)

**Keywords:** influenza, SARS-CoV-2, COVID-19, vaccine, intranasal, mucosal, IgA, live, vector, M2, single-replication, combination

## Abstract

Current SARS-CoV-2 vaccines provide protection for COVID-19-associated hospitalization and death, but remain inefficient at inhibiting initial infection and transmission. Despite updated booster formulations, breakthrough infections and reinfections from emerging SARS-CoV-2 variants are common. Intranasal vaccination to elicit mucosal immunity at the site of infection can improve the performance of respiratory virus vaccines. We developed SARS-CoV-2 M2SR, a dual SARS-CoV-2 and influenza vaccine candidate, employing our live intranasal M2-deficient single replication (M2SR) influenza vector expressing the receptor binding domain (RBD) of the SARS-CoV-2 Spike protein of the prototype strain, first reported in January 2020. The intranasal vaccination of mice with this dual vaccine elicits both high serum IgG and mucosal IgA titers to RBD. Sera from inoculated mice show that vaccinated mice develop neutralizing SARS-CoV-2 antibody titers against the prototype and Delta virus strains, which are considered to be sufficient to protect against viral infection. Moreover, SARS-CoV-2 M2SR elicited cross-reactive serum and mucosal antibodies to the Omicron BA.4/BA.5 variant. The SARS-CoV-2 M2SR vaccine also maintained strong immune responses to influenza A with high titers of anti H3 serum IgG and hemagglutination inhibition (HAI) antibody titers corresponding to those seen from the control M2SR vector alone. With a proven safety record and robust immunological profile in humans that includes mucosal immunity, the M2SR influenza viral vector expressing key SARS-CoV-2 antigens could provide more efficient protection against influenza and SARS-CoV-2 variants.

## 1. Introduction

Since the first reported case in January 2020, the SARS-CoV-2 virus has spread rapidly through the human population, resulting in a coronavirus disease (COVID-19) pandemic responsible for more than 1 million deaths in the United States and over 6.5 million worldwide [1]. Globally, the COVID-19 pandemic caused the worst economic crisis since the Great Depression, increasing poverty and widening economic disparities, especially in developing countries [2,3,4]. Intramuscular (IM) vaccines against SARS-CoV-2, including the first-to-market mRNA and adenovirus-vectored vaccines, induced favorable serum responses which provided greater than 90% vaccine effectiveness in combatting severe disease and reducing COVID-19-associated hospitalizations and deaths [5,6,7]. However, numerous countries, including the United States, have continued to experience multiple peaks in COVID-19 cases, likely due to the emergence of new immune-evading variants, such as Delta in the summer of 2021 and Omicron at the end of that same year [8]. Multiple booster vaccinations, including new multivalent formulations, are now recommended to combat SARS-CoV-2 variants [9,10]. However, the lack of IM vaccine-induced mucosal responses and low durability have rendered current vaccines ineffective at preventing initial infection and reducing SARS-CoV-2 transmission [11,12]. Thus, breakthrough infections in fully vaccinated individuals and reinfections with emerging variants have become commonplace.

The COVID-19 pandemic was also associated with substantial reductions in the circulation of other seasonal respiratory viruses. The precautions implemented to slow the pandemic, such as masking and social distancing, have been credited for the significant reduction in influenza cases from September 2020 to the end of January 2021 [13]. However, the incidence of circulating influenza cases immediately following the lifting of COVID-19 mandates increased significantly. The 2022 influenza season was the worst season Australia had seen in five years, with reported cases roughly three times higher than their national average. Both northern and southern hemisphere influenza seasons peaked about two months earlier than is common, and influenza-related hospitalization and mortality rates in the United States returned to pre-pandemic levels, with an estimated 18,000 to 55,000 deaths, including 149 pediatric deaths, documented as of May 2023 [13]. High circulating levels of both influenza and SARS-CoV-2 have resulted in increased coinfections, which are known to increase disease length and severity in patients with concurrent influenza and COVID-19 [14,15]. Although vaccination remains the single best method of combating influenza-associated illness, the effectiveness of licensed vaccines is highly variable and has been as low as 19% in the past decade [16]. The likelihood that SARS-CoV-2 will continue to co-circulate seasonally with influenza has only escalated the need for more effective vaccine strategies for both viruses.

We previously described an intranasal (IN) M2-deficient single-replication (M2SR) influenza vaccine that provides heterosubtypic cross-protection in mouse and ferret models [17,18,19]. The M2SR influenza virus does not express the M2 protein, rendering it incapable of replication beyond a single cycle [20,21]. When M2SR infects cells, it directs the synthesis of genomic RNA and mRNA, and the expression of all influenza proteins other than M2, generating a multi-faceted immune response that includes systemic and mucosal antibodies as well as cellular responses. In addition to relevant animal models, H3N2 M2SR has provided protection against infection and disease from a highly drifted H3N2 virus in a Phase 2 human influenza challenge study [22]. Moreover, M2SR elicited significant durable levels of mucosal and systemic neutralizing antibodies from subjects in multiple human clinical trials [22,23,24].

The development of a plasmid-based virus rescue system allowed for the generation of recombinant influenza viruses and, eventually, the establishment of influenza viral vectors expressing foreign sequences [25,26,27,28]. Influenza viral vectors containing antigens from other pathogens, such as *Bacillus anthracis* and HIV, have successfully induced immune responses to those antigens in animal models [29,30]. As an influenza viral vector with a well-documented safety profile, and an IN-delivery system shown to induce robust and durable humoral and mucosal responses, the M2SR vaccine has the potential to provide both protection from drifted influenza strains and a mechanism for the delivery of key foreign antigens directly to the upper respiratory tract.

We describe an H3N2 M2SR influenza vaccine encoding the SARS-CoV-2 S1 spike protein receptor-binding domain (RBD) region (SARS-CoV-2 M2SR) of the prototype SARS-CoV-2 strain and its safety and immunological profile within a mouse model [31]. The intranasal SARS-CoV-2 M2SR elicits systemic and mucosal antibodies against SARS-CoV-2 variants while maintaining protective influenza immune responses; hence, a dual COVID-influenza vaccine.

## 2. Materials and Methods

### 2.1. M2SR Expressing Membrane-Anchored SARS-CoV-2 RBD Antigen

**Figure 1:**
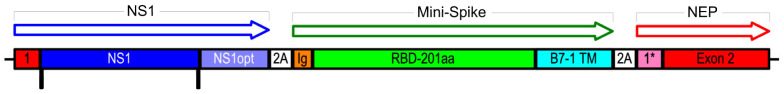
Engineered NS Segment Expressing Tripartite SARS-CoV-2 RBD Fusion Protein. A synthetic ORF encoding SARS-CoV-2 Mini-spike protein [33] (green arrow) composed of Ig heavy chain signal sequence (Ig, orange box), SARS-CoV-2 S1 RBD (RBD-201aa, green box) and murine B7 transmembrane domain (B7-1 TM, turquoise box) was inserted into influenza segment 8 between the NS1 and NEP ORF flanked by 2A self-cleavage sites (2A, white boxes). Segment 8 splicing was abolished by point mutations to splice acceptor and donor sites (black bars). Portions of segment 8 comprising the C-terminus of NS1 and exon 1 of NEP were duplicated, and codon was optimized (NS1opt, light blue; 1*, light red; boxes), restoring expression of full-length NS1 (blue arrow) and essential NEP (red arrow) proteins. The remainder of the NS1 gene (NS1, dark blue box), including former NEP exon 1 (1, red box) and the NEP gene (Exon 2, large red box), was not altered. The engineered segment 8 expresses a single ORF that is processed into 3 functional proteins: NS1, NEP and a membrane-anchored SARS CoV-2 RBD antigen.

A synthetic ORF (IDT, Coralville, IA, USA) encoding the SARS-CoV-2 RBD (RBD) antigen composed of the Ig heavy chain signal sequence, SARS-CoV-2 S1 RBD (Genbank accession MN908947.3) and the murine B7 transmembrane domain flanked by 2A self-cleavage sites was inserted into M2SR NS segment 8 in-frame between the NS1 and NEP ORF [32,33]. Segment 8 splicing was abolished by the introduction of silent point mutations to the acceptor and donor sites. The portions of segment 8 comprising the C-terminus of NS1 and exon 1 of NEP open reading frames (ORF) were duplicated, and the codon was optimized to restore the expression of the full-length NS1 and essential NEP proteins. The modified segment 8, called NS1XS, is designed to express a single ORF that is translated into 3 functional proteins: NS1, NEP and a membrane-anchored RBD vaccine antigen (Figure 1).

### 2.2. SARS-CoV-2 M2SR and Sing2016 M2SR Vaccine Viruses

The SARS-CoV-2 M2SR virus was generated by plasmid-based virus rescue using the modified NS segment encoding RBD, hemagglutinin (HA) and neuraminidase (NA) plasmids derived from the A/Singapore/INFIMH-16-0019/2016 H3N2 (Sing2016) virus, and the remaining segments from the M2SR A/PR/8/1934 backbone, as described previously [17]. Sing2016 M2SR, encoding H3 HA and N2 NA from Sing2016, was described previously [34]. Viruses were amplified in M2VeroA cells, and RT-PCR products corresponding to HA, NA, M2SR and NS1XS were sequenced for identity confirmation.

### 2.3. Production of Vaccines

M2VeroA cells were grown in a 1 L culture vessel format (CELLSTACK 5 chamber, Corning, Corning, NY, USA) infected by SARS-CoV-2 M2SR or Sing2016 M2SR (MOI = 0.001) in a chemically defined OptiVERO (Invitria) medium containing 1.1 µg/mL recombinant trypsin (Roche, Indianapolis, IN, USA) and incubated at 35 °C for 3 days. Clarified supernatant sterile filtered through a 0.2 µm aPES membrane filter was subjected to benzonase nuclease digestion. Culture fluid was concentrated and diafiltered by tangential flow filtration (TFF) using a MidiKros 300,000 MWCO mPES hollow fiber filter (Repligen, Boston, MA, USA) into sucrose phosphate glutamate (SPG) buffer with a pH of 7.2 [24], and ultracentrifuged at 110,000× *g* through 25% sucrose in PBS for 2 h. The virus pellet was resuspended in SPG and stored frozen at −80 °C. The virus titer was measured by 50% tissue culture infectious dose assay (TCID_50_), defined as the hemagglutination assay (HA) positivity of virus culture in M2CK cells, using the method of Reed and Muench [20,35].

### 2.4. Immunoblot Analysis

M2VeroA cells infected with SARS-CoV-2 M2SR, Sing2016 M2SR virus at MOI = 5 or media (mock) or purified M2SR preparations were lysed (1X LDS, ThermoFisher Scientific, Waltham, MA, USA) under reducing conditions in the presence of Benzonase nuclease (Sigma-Aldrich, St. Louis, MO, USA). Infected cell lysates prepared at 24 h post-infection or disrupted virions were separated by polyacrylamide gel electrophoresis under denaturing and reducing conditions, transferred to nitrocellulose membrane and probed with rabbit polyclonal antibodies directed against SARS-CoV-2 Spike S1 RBD (Sino Biological, Wayne, PA, USA) or influenza Sing2016 H3N2 HA, followed by an anti-rabbit IgG HRP conjugate (Southern Biotech, Birmingham, AL, USA). Bands were visualized using 1-Step Ultra TMB-Blotting Solution (ThermoFisher).

### 2.5. Immunofluorescence Microscopy of SARS-CoV-2 RBD and Sing2016 H3N2 HA

Vero (CCL-81, ATCC, Manassas, VA, USA) and MDCK (ECAAC 84121903, Sigma-Aldrich, St. Louis, MO, USA) cells infected with SARS-CoV-2 M2SR, Sing2016 H3N2 M2SR (MOI = 5) or media (mock) were fixed at 24 h post-infection with 4% formalin, and then incubated with the human anti-SARS-CoV RBD monoclonal CR3022 (Abcam, Boston, MA, USA) or mouse anti-H3 HA monoclonal (NR-52364, BEI, Manassas, VA, USA) antibody, followed by secondary antibody goat anti-human IgG Alexa Fluor 488 or goat anti-mouse IgG Alexa Fluor 647 conjugates (Southern Biotech, Birmingham, AL, USA). Labeled cells were stained with DAPI and visualized using an ECHO fluorescent microscope on FITC, Cy5 and DAPI channels, respectively.

### 2.6. Flow Cytometric Analysis of SARS-CoV-2 RBD and Sing2016 H3N2 HA Cell Surface Expression

Vero and MDCK cells were infected in TC12 wells, as described above for microscopy. After 24 h, the cells were dissociated using Cell Stripper reagent (Corning, Corning, NY, USA) and washed twice in FACS buffer (2% FBS in DPBS). Live, intact cells in FACS buffer at 4 °C were surface labeled for SARS-CoV-2 RBD and H3 expression, as described for immunofluorescence labeling. RBD and HA surface expression was assessed by measuring fluorescence at 488 nm and 647 nm, respectively, using a BD FACSymphony A3 instrument (BD Biosciences, San Jose, CA, USA). Data were analyzed using FlowJo software version 10.8.1. (FlowJo, Ashland, OR, USA).

### 2.7. Mouse Studies

Female BALB/c, 6- to 7-week-old mice (Inotiv, Madison, WI, USA) were used for this study, and the minimum number of animals necessary to identify significant differences between groups via ANOVA was calculated using the Resource equation method [36,37]. All animal study protocols were approved by the FluGen Institutional Animal Care and Use Committee and all experiments were performed in accordance with the National Institute of Health guidelines for the care and use of laboratory animals. Groups of 5 mice were anesthetized with isofluorane and immunized intranasally (IN) with two doses, 21 days apart, of either 10^6^ (Low) or 10^7^ (High) TCID_50_ SARS-CoV-2 M2SR or Sing2016 H3N2 M2SR. Since the manufacturing of on-market COVID mRNA vaccines is currently limited to FDA-approved use in humans, we were not able to secure them for our study. As an alternative, a plasmid vector encoding the entire SARS-CoV-2 Spike Glycoprotein was obtained from BEI and used as a positive control in our study. Specifically, the positive control groups were immunized intramuscularly (IM) with 2 doses of 100 µg/mouse vector pCAGGS containing SARS-CoV-2 Spike Glycoprotein DNA (BEI, Manassas, VA, USA), and the mock vaccination groups received 2 doses IN of SPG buffer alone. Animal body weight was monitored at least 4 days prior and at least 9 days post-immunization. Serum was collected on Days −5, 19 and 42, and trachea-lung washes were collected from each mouse on day 42.

### 2.8. Enzyme-Linked Immunosorbent Assay (ELISA)

Immunoglobulin G (IgG) and IgA antibody titers were measured in the sera and trachea-lung washes of immunized mice by use of an enzyme-linked immunosorbent assay (ELISA). The ELISA was performed using the soluble Sing2016 H3N2 HA protein (Sino Biological US, Houston, TX, USA) or SARS-CoV-2 Spike RBD protein (BA.4/BA.5) with C-terminal HIS-tag (Sino Biological) or SARS-CoV-2 Spike RBD protein (Genbank accession MN908947.3) with a C-terminal HIS-tag (NR-52309, BEI, Manassas, VA, USA) transiently expressed in 293T cells and purified by using COMPLETE™ His-Tag Purification resin (Roche, Basel, Switzerland). The 96-well immunoplates (Nunc-Maxisorp; Nunc A/S, Roskilde, Denmark) were coated overnight at 4 °C with 100 μL of the HA or RBD protein at a concentration of 2 μg/mL in phosphate-buffered saline (PBS). After blocking the plate with PBS containing 0.1% polysorbate 20 (PBS-T) and 5% non-fat dried milk (NFDM), the plates were incubated in duplicate with mouse serum or trachea-lung wash diluted in PBS-T with 0.5% of NFDM. After a two-hour incubation at room temperature, the plates were washed with PBS-T three times and then incubated with either anti-mouse IgG or anti-mouse IgA secondary antibody conjugated with horseradish peroxidase (KPL Seracare, Milford, MA; 1:2000 dilution in PBS-T with 0.5% NFDM). The plates were washed six times with PBS-T, developed with 1-STEP™ Ultra TMB-ELISA Substrate Solution (Thermo Fisher Scientific, Waltham, MA, USA) and stopped with 4N sulfuric acid. The absorbance was measured at a wavelength of 450 nm (OD450) using a ChroMate 4300 microplate reader (Awareness Technology, Palm City, FL, USA). The endpoint titers were the reciprocal of the dilution that was above the OD450 cut-off value of 0.3.

### 2.9. Hemagglutination Inhibition Assay (HAI)

Serum samples from immunized mice were evaluated by hemagglutination inhibition assay (HAI). Serum samples were pooled by group and treated with a receptor-destroying enzyme (Denka-Seiken, Tokyo, Japan) overnight at 37 °C, followed by heat inactivation for 1 h at 56 °C. Two-fold serially diluted serum was incubated with 8 HA units/50 μL of Sing2016 H3N2 virus at ambient temperature for 30 min, and then incubated with 0.5% turkey red blood cells (Innovative Research, Novi, MI, USA) for an additional 30 min. The HAI titer is the reciprocal of the highest dilution of serum that prevented hemagglutination.

### 2.10. Plaque Reduction Neutralization Titer (PRNT)

Neutralizing antibody levels were determined by SARS-CoV-2 PRNT under BSL3 containment, as described previously [38]. Briefly, sera were first heat-inactivated for 30 min at 56 °C, then a two-fold dilutions series in BA-1 media (Tris-buffered MEM containing 1% BSA) was prepared in a 96-well plate starting at a 1:5 dilution. Serum from a cat experimentally infected with SARS-CoV-2 WA1 was used as a positive control. Pooled human sera taken from patients who received 1 and 2 doses of Moderna’s mRNA vaccine were also tested (NRH-17846, NRH-21747 BEI, Manassas, VA, USA). An equal volume SARS-CoV-2 virus (isolates USA-WA1/2020 or delta (B.1.617.2) USA/PHC658/2021) was added to the serum dilutions and the sample-virus mixture was gently mixed. Following incubation at 37 °C for 1 h, serum-virus mixtures were plated onto Vero E6 cell monolayers in 6-well tissue culture plates. The plates were rocked every 10–15 min for 45 min and then overlaid with 0.5% agarose in MEM without phenol red and incubated for 1 d at 37 °C, 5% CO_2_. A second overlay with neutral red dye was added at 24–30 h, and the plaques were counted at 48–72 h post-plating. The antibody titers were recorded as the reciprocal of the highest dilution in which >80% of the virus was neutralized.

### 2.11. Statistical Analyses

Statistical analyses, as described in the figure legends, were performed using GraphPad Prism version 9.4. (GraphPad Software, San Diego, CA, USA).

## 3. Results

### 3.1. Generation of SARS-CoV-2 H3N2 M2SR Vaccine

The previously described A/Singapore/INFIMH-16-0019/2016 H3N2 (Sing2016 H3N2) M2SR influenza vaccine virus was modified to also encode a membrane-anchored form of the receptor binding domain (RBD) of the SARS-CoV-2 spike to generate a dual COVID/influenza vaccine candidate, SARS-CoV-2 M2SR [23,33,39].

The M2SR NS segment 8 was engineered (NS1XS) to express a single ORF that is translated into three functional proteins: NS1, NEP and a membrane-anchored SARS-CoV-2 RBD (Figure 1). The expression of SARS-CoV-2 RBD was achieved via the fusion of the prototype S1 RBD coding sequence to the full-length NS1 open reading frame (ORF), separated by a viral 2A site that allows for the expression of separate NS1 and SARS-CoV-2 peptides. The expression of the essential NEP nuclear export function was maintained by the insertion of a second 2A site after the NS1 and RBD genes to allow for the translation of functional in-frame NEP ORF, starting with proline. Remnant 14 amino acids of the 2A sites are appended to the C-termini of both the NS1 and RBD proteins.

### 3.2. Growth Kinetics of SARS-CoV-2 M2SR in Tissue Culture

**Figure 2:**
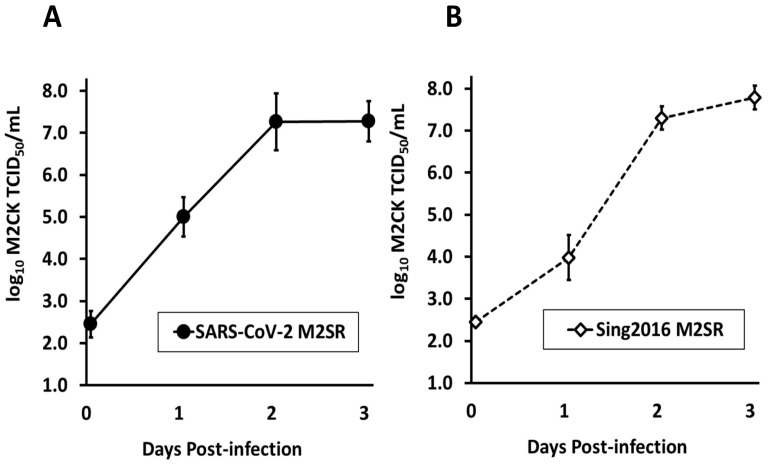
Growth kinetics of SARS-CoV-2 M2SR and Sing2016 H3N2 M2SR vector. M2VeroA cells propagated under animal origin-free (AOF) conditions suitable for human vaccine manufacture were inoculated at MOI = 0.001 with M2SR viruses. (**A**) SARS-CoV-2 M2SR vaccine candidate (filled circle, solid black line) was in 5 independent 1 L culture vessels (N = 5). (**B**) Control data from three independent triplicate inoculations (5 mL vessel) with Sing2016 M2SR vector only (open diamond, dotted black line) were compiled (N = 9) for comparison. Culture supernatant was sampled daily and stored frozen for analysis of titer by TCID_50_ assay in M2CK cells. Day 0 TCID_50_/mL value is TCID_50_ of inoculum in volume of virus culture. Plots of growth rate for the two strains given as mean daily titer and standard deviation vs. time in culture are comparable.

A bench scale (5 L) simulated manufacture of SARS-CoV-2 M2SR was performed by the infection of M2VeroA production cells (MOI = 0.001) in five 1 L (5-stack) culture vessels under animal origin-free (AOF) culture conditions. The cultures were sampled daily for 3 d and the virus titer was determined by TCID_50_ assay. The growth kinetics of SARS-CoV-2 M2SR and Sing2016 M2SR were comparable. SARS-CoV-2 M2SR reached peak titer on day 3 of log_10_ 7.28 ± 0.48 TCID_50_/mL, similar within a standard deviation to the Sing2016 M2SR influenza virus, which achieved a day 3 peak titer of 7.79 ± 0.28 TCID_50_/mL (Figure 2), indicating that the M2SR virus is tolerant of the RBD insertion. These results indicate that SARS-CoV-2 M2SR growth is comparable to the Sing2016 M2SR vaccine vector only.

### 3.3. RBD Expression in SARS-CoV-2 M2SR-Infected Cells and Virions

**Figure 3:**
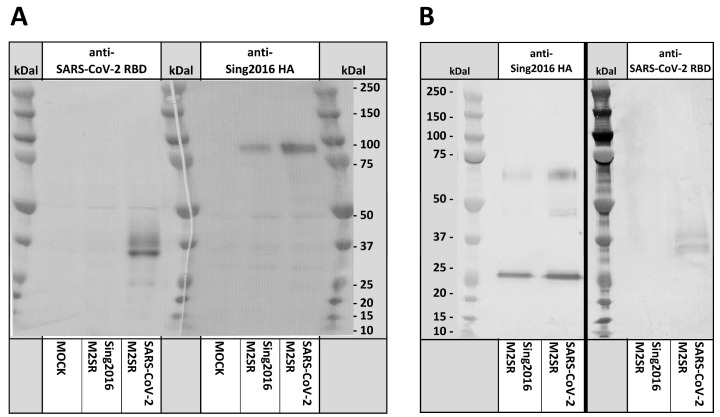
H3 and RBD Immunoblot Analysis. (**A**) Immunoblot of SARS-CoV-2 M2SR and Sing2016 M2SR-infected Vero Cells. M2VeroA cells grown for 24 h in AOF media in TCT TC6-well to 90% confluency were inoculated at MOI = 5 in the absence of trypsin by SARS-CoV-2 M2SR, with Sing2016 M2SR vector virus, and mock media only controls. After 24 h, whole cells were lysed directly from the TC6-well, reduced and denatured prior to SDS-PAGE. Resolved proteins were transferred to replicate nitrocellulose membranes for immunoblot analysis with rabbit polyclonal antisera directed against SARS-CoV-2 RBD (left panel) and Sing2016 H3 hemagglutinin (HA) from influenza Sing2016 H3N2 (right panel). Specific Ab–Ag interactions were detected using an anti-rabbit IgG HRP conjugate detected by colorimetric substrate TMB. (**B**) SARS-CoV-2 M2SR and Sing2016 M2SR Virions. Purified virus preparations were normalized to a HA titer of 3200, a viral titer of 3 × 10^9^ TCID_50_/mL. Viruses were lysed, reduced and denatured prior to SDS-PAGE. Resolved proteins were transferred to replicate nitrocellulose membranes for immunoblot analysis with rabbit polyclonal antisera directed against Sing2016 H3 (left panel) hemagglutinin from influenza Sing2016 H3N2 and SARS-CoV-2 RBD (right panel). Specific Ab–Ag interactions were detected using anti-rabbit IgG HRP conjugate detected by colorimetric substrate TMB.

M2VeroA cells were infected with purified SARS-CoV-2 M2SR and Sing2016 M2SR viruses at MOI = 5 or mock-infected with media only. After 24 h, total cell protein extracts were prepared for immunoblot analysis using polyclonal antisera against SARS-CoV-2 S1 RBD or Sing2016 HA. The anti-SARS-CoV-2 RBD antibody detected a strong 32 kD band and a weaker 37 kD band in SARS-CoV-2 M2SR-infected M2VeroA, but not in Sing2016 M2SR-infected or mock-infected cells (Figure 3A). The 32.2 kD band corresponds to the predicted size of the RBD B7.1 TM fusion protein. The 37 kD band likely reflects RBD glycosylation, suggesting that the fusion protein is transported via the secretory pathway as intended (Figure 3A). Partial cleavage at the 2A sites is not likely, as that scenario would result in fusion proteins of either 46.4 kD (RBD-NEP fusion) or 62.0 kD (NS1-RBD fusion), which are larger than the observed 37 kD RBD-sera-reactive protein.

The anti-Sing2016 HA sera detected an 85 kD band corresponding to the glycosylated hemagglutinin HA0 (predicted MW 63.7 kD) in both SARS-CoV-2 M2SR and Sing2016 M2SR-infected cells, but not in the mock-infected cells. The intensity of staining for the detected H3 HA protein bands appears to be similar between the two strains. These results suggest that the expression of the RBD does not impact HA expression or transport to the cell membrane.

We next tested the composition of purified M2SR virions using H3 and RBD immunoblots. Both SARS-CoV-2 M2SR and Sing2016 M2SR contain 2 major bands that are detected by anti-Sing2016 HA antisera. These bands with an apparent MW of about 68 kDal and 27 kDal correspond to the HA1 and HA2 trypsin cleavage products expected from H3. Surprisingly, the anti-RBD serum was able to detect the RBD B7.1 TM fusion protein from extracts of the highly purified SARS-CoV-2 M2SR virions (Figure 3B). In addition, the 37 kDal MW observed for the RBD serum reactive band is the same as that detected from infected Vero cell extracts. Thus, it appears that at least a portion of the RBD antigen fusion protein is incorporated into the SARS-CoV-2 M2SR virions. No RBD antigen was detected in Sing2016 M2SR control virus extracts.

### 3.4. Sub-Cellular Detection of RBD Expression by Immunofluorescence

**Figure 4:**
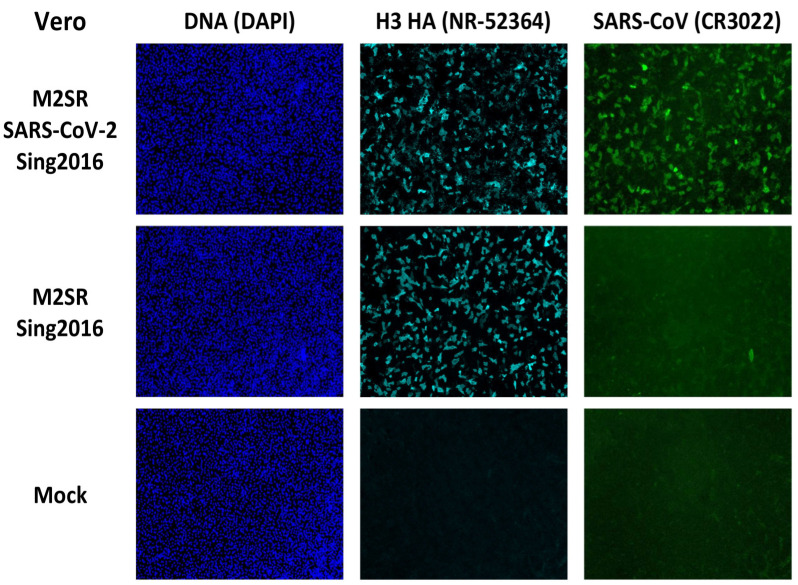
Immunofluorescence microscopy of SARS-CoV-2 RBD and Hemagglutinin H3 in M2SR-infected Vero Cells. Vero cells cultured for 24 h in 10% FBS-DMEM/F12 media were inoculated at MOI = 5 with either SARS-CoV-2 M2SR encoding RBD (top row) or Sing2016 M2SR vector only (middle row). Mock infection was media only (bottom row). At 24 h post-inoculation, cells were fixed with paraformaldehyde and stained with DAPI (DNA, left column), mouse anti-H3 mAb (BEI NR-52364, center column) or human anti-SARS-CoV Spike RBD mAb (CR3022, right column). Secondary Abs were Alexa 647 labeled anti-mouse IgG and Alexa 488 labeled anti-human IgG. Both the Sing2016 M2SR vector and SARS-CoV-2 M2SR (Sing2016)-infected cells have robust H3 staining as compared to mock, while only SARS-CoV-2 M2SR-infected cells exhibit specific staining for SARS-CoV-2 RBD. DAPI stain demonstrates intact cell nuclei remain under all conditions.

To visualize the sub-cellular location of the SARS-CoV-2 RBD antigen expressed after infection with the SARS-CoV-2 M2SR vaccine, Vero and MDCK cells cultured in chamber slides (LabTek) were infected by M2SR viruses at MOI = 5 or mock-infected with media and incubated for 24 h prior to formalin fixation. Fixed cells were incubated with a neutralizing human anti-SARS-CoV RBD monoclonal (CR3022) or mouse anti-H3 hemagglutinin ascites followed by fluorescently labeled goat anti-human IgG Alexa Fluor 488 conjugate or goat anti-mouse IgG Alexa Fluor 647 conjugates. Immunostained cells were counterstained with DAPI to detect cell nuclei and imaged using three-color fluorescence microscopy (DAPI, Cy5, and FITC; Figure 4). The DNA staining by DAPI shows multiple intact cell nuclei after the three infections: SARS-CoV-2 M2SR, Sing2016 M2SR vector and mock. Anti-H3 HA antisera detected strong, comparable HA expression in both SARS-CoV-2 M2SR and Sing2016 M2SR-infected cells, and not in mock-infected cells. While the bright H3-associated fluorescence seen along the cell margins is expected for a cell membrane antigen, cytoplasmic staining was also seen, suggesting that expressed HA is in transit to the cell surface within 24 h under these virus culture conditions.

The anti-SARS-CoV RBD monoclonal detected strong fluorescence that co-localizes to cells expressing HA in SARS-CoV-2 M2SR-infected cells, but not in Sing2016 M2SR- or mock-infected cells. Similar to HA expression, the RBD protein was significantly localized to the cell membrane, although cytoplasmic staining was also apparent. These results show that HA trafficking is not affected in the SARS-CoV-2 M2SR-infected cells, and the RBD fusion protein travels to the membrane as intended.

### 3.5. RBD Surface Expression Detection by Flow Cytometry

**Figure 5:**
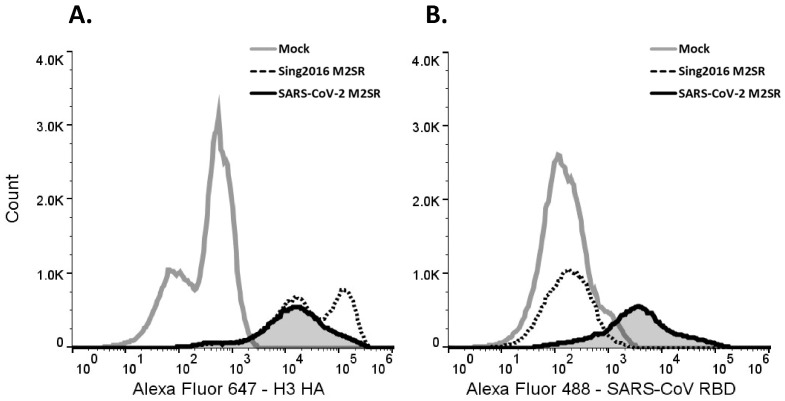
Flow cytometry of SARS-CoV-2 RBD and H3 expression from infected Vero cells. Vero cells were inoculated at MOI = 5 by SARS-CoV-2 M2SR (solid black line) and Sing2016 M2SR vector alone (dotted black line) viruses, or mock media only (solid gray line). Live cells were surface labeled for both (**A**) Sing2016 H3 hemagglutinin with mouse anti-H3 hemagglutinin mAb and (**B**) SARS-CoV RBD by human mAb CR3022 for 24 h post-inoculation. Cell size parameters and fluorescence at 488 nm and 647 nm were obtained by flow cytometry.

The observation that the SARS-CoV-2 M2SR vaccine candidate can direct the cell surface expression of the RBD antigen in Vero cells was confirmed by the flow cytometry of Vero cells. Intact live cells were stained with RBD- or H3-specific mAbs and labeled secondary reagents. A histogram analysis of SARS-CoV-2 M2SR and Sing2016 M2SR inoculated Vero cells demonstrated comparable HA surface expression, but only SARS-CoV-2 M2SR demonstrated cell surface expression of the RBD fusion protein (Figure 5). As expected, mock-infected cells did not display expression of either HA or RBD. Thus, the SARS-CoV-2 M2SR virus directs cell surface expression of the membrane-associated SARS-CoV-2 RBD antigen.

### 3.6. Evaluation of In Vivo Immune Responses Induced by SARS-CoV-2 M2SR Vaccine

**Figure 6:**
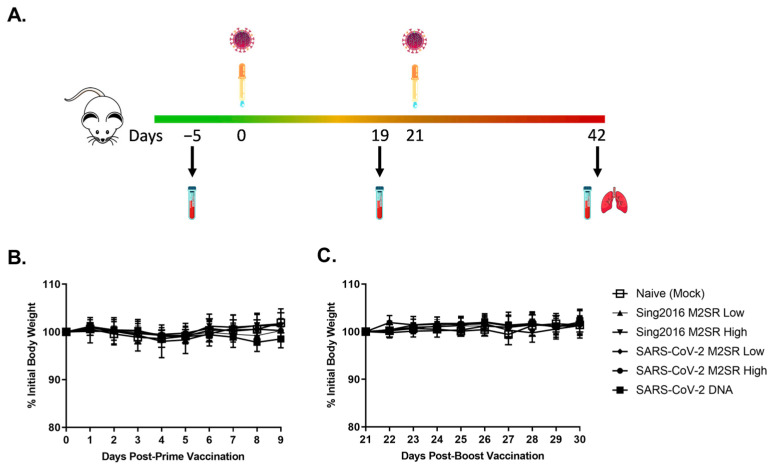
Vaccination scheme, sampling regimen and body weight changes in mice vaccinated with SARS-CoV-2 M2SR. (**A**) Six groups of BALB/c mice (N = 30) were vaccinated with 10^6^ (Low) or 10^7^ (High) TCID_50_ of Sing2016 H3N2 M2SR vector only, SARS-CoV-2 M2SR, SARS-CoV-2 DNA or SPG buffer as naïve (mock) on Day 0 (Prime) and again on Day 21 (Boost). Serum samples were collected on Days −5, 19 and 42. Lung wash samples were also collected on Day 42 for each group. Mouse body weights were measured daily from at least 4 days prior to and at least 9 days following prime (**B**) and boost (**C**) vaccinations. The mean body weight of the individual mice measured for four days prior to vaccination was used as 100% body weight to normalize the data. Mean % body weight and SD for each group is shown.

Mice were intranasally inoculated on study day 0 (prime) and 21 (boost) with either 10^6^ (Low) or 10^7^ (High) TCID_50_ of Sing2016 M2SR vector only, SARS-CoV-2 M2SR or buffer control (mock) (Figure 6A). One group of mice was also administered a prime and boosting dose of SARS-CoV-2 DNA intramuscularly. No mice exhibited signs of illness and there was no significant difference in body weights between Sing2016 M2SR vector only, SARS-CoV-2 M2SR and mock-inoculated mice (Figure 6B), suggesting this novel vaccine candidate is well-tolerated and non-pathogenic in vivo.

**Figure 7:**
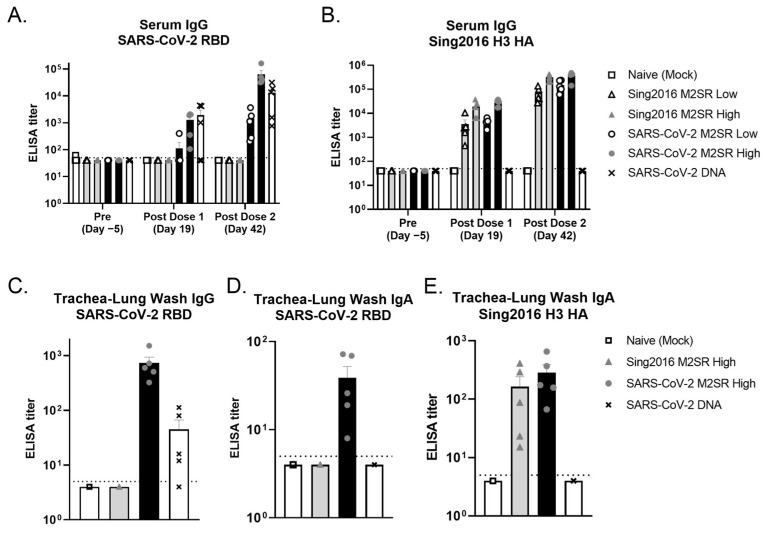
Humoral and mucosal antibody responses in mice vaccinated with SARS-CoV-2 M2SR. BALB/c mice were immunized, as described in the Section 2, and serum was collected on Days −5, 19 and 42. (**A**) Anti-SARS-CoV-2 RBD and (**B**) Anti-Sing2016 H3N2 HA immunoglobulin G (IgG) in individual mouse serum was detected by ELISA. Trachea-lung washes were collected from each mouse on Day 42. (**C**) Anti- SARS-CoV-2 RBD IgG, (**D**) Anti- SARS-CoV-2 RBD immunoglobulin A (IgA) and (**E**) Anti-Sing2016 H3 IgA in individual trachea-lung wash was also detected by ELISA. Endpoint antibody titers (mean ± SD) are given as the reciprocal of the dilution determined by the least squares method, giving an OD reading of 0.3 at 450 nm. Detection limits of 50 for serum IgG and 5 for trachea-lung wash IgA and IgG are indicated with dotted lines.

Serum samples were collected pre-study and following both prime and boost inoculations (Figure 6A). As shown in Figure 7A, serum SARS-CoV-2 IgG levels specific for prototype RBD were significantly increased following a single inoculation with SARS-CoV-2 M2SR. This increase was dose-dependent, and both low and high dose groups experienced enhanced immunogenicity following a second inoculation. SARS-CoV-2 M2SR-induced RBD IgG levels were comparable to that of intramuscularly delivered SARS-CoV-2 DNA, following both prime and boost immunizations. Sing 2016 HA-specific serum IgG levels pre- and post-inoculation were also evaluated for each group. Comparable, dose-dependent increases in IgG were seen both in Sing2016 M2SR vector only and in SARS-CoV-2 M2SR groups following one and two doses, while the naïve mock and SARS-CoV-2 DNA group IgG remained below the level of detection at all three time points (Figure 7B).

To assess local mucosal immune responses, trachea-lung washes were collected from each mouse post-study and evaluated for antigen-specific IgG and IgA. In Figure 7C,D, SARS-CoV-2 RBD-specific IgG and IgA levels were significantly increased in all animals that received SARS-CoV-2 M2SR, as compared to naïve and Sing2016 M2SR vector controls. In contrast, the lung wash from animals that received SARS-CoV-2 DNA intramuscularly had no appreciable RBD IgA. Furthermore, Sing2016 H3N2 HA-specific IgA found in trachea-lung washes following inoculation with Sing2016 M2SR vector only and SARS-CoV-2 M2SR were comparable and significantly increased over naïve controls or those that received SARS-CoV-2 DNA (Figure 7D). These data indicate that the vaccine candidate SARS-CoV-2 M2SR stimulates both systemic and mucosal immune responses to both SARS-CoV-2 RBD and influenza HA.

### 3.7. SARS-CoV-2 M2SR Vaccine Induces Functional Antibodies against Influenza and SARS-CoV-2 Variants

**Figure 8:**
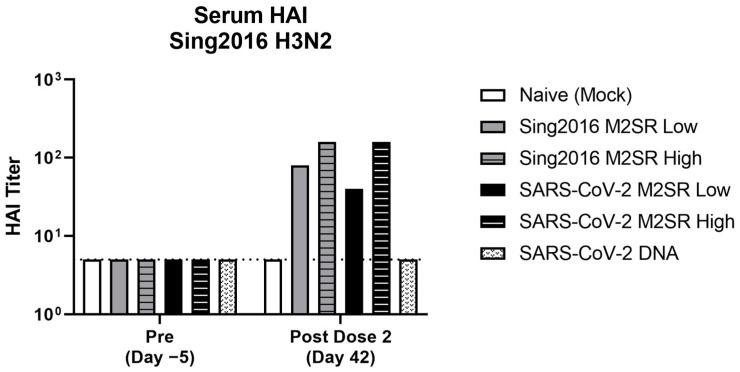
Serum HAI antibody responses to A/Singapore/INFIMH-16-0019/2016 H3N2. Serum samples were collected from immunized mice pre- (Day −5) and post- (Day 42) study. Hemagglutination assay inhibition (HAI) titers against Sing2016 H3N2 virus were determined for pooled RDE-treated sera per group. HAI titers are expressed as the reciprocal of the highest dilution of serum inhibiting agglutination of 0.5% of turkey erythrocytes at 8 HA units per 50 µL with lower limit of detection of 5 per 50 µL (indicated with dotted line).

To evaluate the functional immunogenicity of the influenza and SARS-CoV-2 specific antibodies produced following SARS-CoV-2 M2SR vaccination, an influenza hemagglutination inhibition (HAI) assay and a SARS-CoV-2 viral plaque reduction neutralization test (PRNT_80_) were performed. Pre-study (Day −5) and post-dose 2 (Day 42) sera were collected from each group and pooled, and HAI titers were determined against the Sing2016 H3N2 virus. As expected, pre-study serum HAI values against the Sing2016 H3N2 antigen were below the baseline for all groups (Figure 8). Post-vaccination values for M2SR vector only and M2SR SARS-CoV-2 were comparable and significantly enhanced compared to both the naïve and SARS-CoV-2 DNA control.

**Figure 9:**
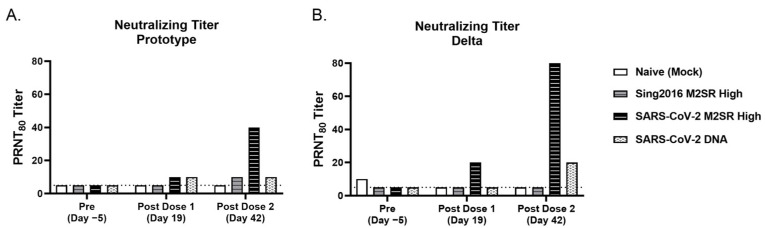
Serum neutralizing antibody responses to variants of SARS-CoV-2. Serum samples were collected from immunized mice pre-study (Day 5), post-prime (Day 19) and post-boost (Day 42). Neutralization activity of the sera against 2 SARS-CoV-2 virus isolates (**A**) Prototype (USA-WA1/2020) or (**B**) Delta (B.1.617.2 USA/PHC658/2021) was determined on pooled sera from each group. Antibody titers were recorded as the reciprocal of the highest dilution in which >80% of virus was neutralized. Assay limit of detection given by dotted line.

Viral neutralization titers against two SARS-CoV-2 isolates, USA-WA1/2020 (prototype) or B.1.617.2 USA/PHC658/2021 (Delta), were also determined in pooled, pre-study, post-dose 1 and post-dose 2 serum samples from each group. As predicted, all serum collected pre-study exhibited PRNT_80_ titers at or slightly above the baseline for both SARS-CoV-2 variants (Figure 9). Titers slightly above the baseline for some groups, such as PBS, may be attributed to low level non-specific antibody cross-reactivity. For the 2020 isolate (Figure 9A), PRNT_80_ titers were increased following inoculation with SARS-CoV-2 M2SR, while titers for naïve or M2SR vector only groups remained at or near the baseline. Mice that received SARS-CoV-2 M2SR achieved enhanced neutralizing titers following a second inoculation. Interestingly, SARS-CoV-2 M2SR-induced PRNT_80_ titers were comparable to those of intramuscularly delivered SARS-CoV-2 DNA following prime immunization, but were significantly elevated following boost, while the DNA group exhibited no boosting effect. In Figure 9B, the results were similar with the Delta SARS-CoV-2 isolate, except the SARS-CoV-2 M2SR group was the only group above the baseline following the first vaccine dose, and the titers following the prime and boost vaccinations from this group were further elevated above those seen with the prototype isolate. Pooled human sera taken from individuals who received one and two doses of Moderna’s mRNA vaccine were used as assay controls, and the PRNT_80_ titers against the Delta isolate were 80 and 160, respectively.

### 3.8. Evaluation of SARS-CoV-2 M2SR-Induced Antibody Response to SARS-CoV-2 Omicron Variant

**Figure 10:**
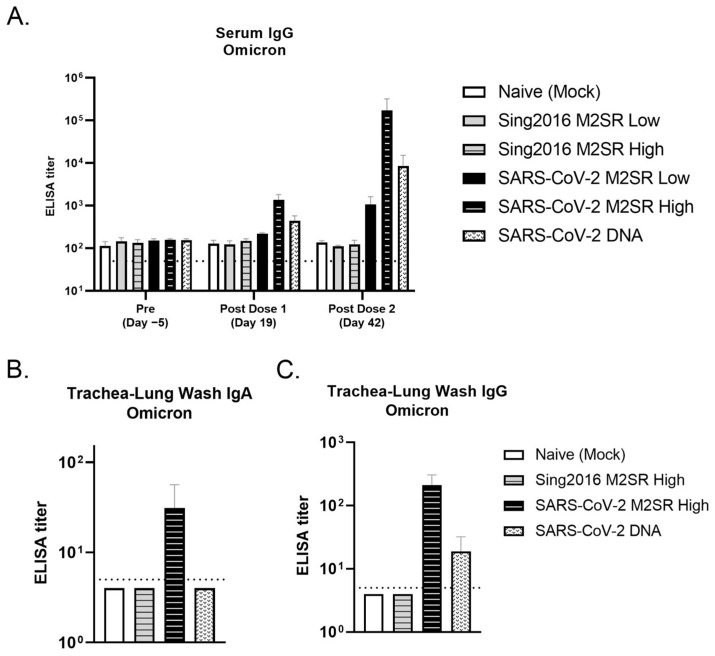
Humoral and mucosal antibody responses to Omicron SARS-CoV-2 RBD in mice vaccinated with SARS-CoV-2 M2SR. BALB/c mice were immunized as described in the Section 2. Serum was collected on Days −5, 19 and 42. (**A**) Anti-Omicron SARS-CoV-2 RBD IgG in individual mouse serum was detected by ELISA. Trachea-lung washes were collected from each group on Day 42, and Anti-Omicron SARS-CoV-2 RBD IgA (**B**) and IgG (**C**) were also detected by ELISA. Endpoint antibody titers (mean ± SD) are given as the reciprocal of the dilution determined by the least squares method, giving an OD reading of 0.3 at 450 nm. Detection limits were 50 for serum IgG and 5 for trachea-lung wash IgA and IgG (indicated with dotted lines).

To further evaluate the breadth of SARS-CoV-2 M2SR-induced antibody responses, sera and trachea-lung washes were collected from each group and evaluated for cross-reactivity with the SARS-CoV-2 Omicron BA.4/BA.5 variant. As shown in Figure 10A, serum IgG levels specific for Omicron RBD were significantly increased following a single high dose inoculation with SARS-CoV-2 M2SR. A dose-dependent increase in IgG levels was observed following SARS-CoV-2 M2SR boost vaccination, and these levels were comparable or superior to those seen from the DNA vaccine control group. The naïve and Sing2016 M2SR control groups remained at or near the baseline.

Mucosal responses to the Omicron RBD variant in trachea-lung washes were also assessed. As seen in Figure 10B, Omicron RBD-specific IgA levels in the trachea-lung wash were measurably increased in mice that received SARS-CoV-2 M2SR. No Omicron specific IgA was detected in mice that received the Sing2016 M2SR control or intramuscular SARS-CoV-2 DNA. The levels of mucosal IgG to Omicron RBD were also increased following SARS-CoV-2 M2SR, reaching 10-fold higher levels than those seen following intramuscular DNA vaccination (Figure 10C). These data indicate that SARS-CoV-2 M2SR stimulates broad systemic and mucosal immune responses that are able to recognize drifted SARS-CoV-2 variants.

## 4. Discussion

Herein, we present evidence that the intranasal influenza vaccine M2SR, designed to express a membrane-anchored spike RBD antigen, is a safe and effective viral vector able to generate humoral and mucosal immune responses to both influenza and SARS-CoV-2 variants in the mouse model.

While approved SARS-CoV-2 vaccines have greatly reduced the risk of severe illness and COVID-19-associated death, current formulations struggle to prevent initial infection and reduce the viral transmission of drifted variants. The COVID-19 pandemic has highlighted the need for mucosal immunity to decrease viral shedding and transmission [40,41]. Animals that received intranasal SARS-CoV-2 M2SR induced measurable systemic and mucosal antibody responses to SARS-CoV-2 RBD. Serum IgG responses were robust and functional, with sera from SARS-CoV-2-vaccinated groups able to effectively neutralize the prototype and Delta variant wildtype virus. The SARS-CoV-2 M2SR was also able to generate robust mucosal IgA and IgG responses to SARS-CoV-2. The cross-reactivity of both serum and mucosal antibodies extended to the drifted Omicron strain, suggesting that SARS-CoV-2 M2SR could provide an increased breadth of protection against emerging SARS-CoV-2 variants. It is noteworthy that like previous testing with parental M2SR, SARS-CoV-2 M2SR was well tolerated in vivo, with no weight loss or clinical signs of illness in mice that received one or two high titer doses, as compared to the mock-infected animals.

Other investigational SARS-CoV-2 vaccines with intranasal delivery systems inducing mucosal immunity have provided superior protection over the primary vaccine series in animal models (see Supplementary Table S1 for studies utilizing similar vaccine strategies) [42,43,44,45,46]. SARS-CoV-2 M2SR maintained effective influenza immunoreactivity while providing strong and specific immune responses to SARS-CoV-2 variants—a distinction from other intranasal vaccine candidates. Mice inoculated with intranasal SARS-CoV-2 M2SR displayed robust serum IgG and mucosal IgA antibodies toward A/Singapore/INFIMH-16-0019/2016 HA at levels equal to or above the Sing2016 M2SR control virus. Functional HAI antibodies reached levels that were considered protective following both low and high doses of SARS-CoV-2 M2SR, and were equivalent to those seen following Sing2016 M2SR immunization, suggesting that the expression of foreign antigens does not impair M2SR-induced immune responses to influenza.

Previously, others have described influenza viral vectors encoding SARS-CoV-2 antigens in place of various influenza genes, including HA, NA and NS1 [33,47,48]. Despite varying degrees of success, it is clear from these constructs that there may be transgene capacity limitations within an influenza vector. Influenza virus nonstructural protein 1 (NS1) is a key virulence factor with established roles in viral replication [49,50]. While the NS1 protein is not essential for viral replication, influenza viruses with truncated or deleted NS1 grow at relatively low levels in key cell types, hampering the commercial development of deleted NS1 live attenuated influenza vaccines (LAIV) [51,52]. Additionally, LAIV candidates based on NS1 deletion have not generated significant seroconversion in human trials, and recently, an NS1-deleted LAIV candidate expressing SARS-CoV-2 antigens was only weakly immunogenic in humans [53,54]. The modification of the NS1 region of M2SR to express SARS-CoV-2 RBD did not attenuate the expression of full-length NS1, and cellular inoculation with SARS-CoV-2 M2SR allowed for the robust targeted membrane expression of the RBD protein. In permissive cells, SARS-CoV-2 M2SR replicated with similar growth kinetics as the unmodified Sing2016 M2SR control virus suggested that M2SR can carry and correctly deliver foreign antigens without being detrimental to current vaccine production methods.

Monovalent M2SR vaccines have demonstrated an ability against multiple subtypes of influenza A and B, and recently we demonstrated the lack of strain interference in mice and ferrets inoculated with quadrivalent M2SR [17,18,19,21]. A quadrivalent M2SR formulation, in which numerous key SARS-CoV-2 antigens were incorporated into each monovalent component, would address any size limitations of the influenza vectored vaccine system and ensure fuller antigenic coverage similar to that provided by full-length spike mRNA vaccines.

## 5. Conclusions

In conclusion, next-generation and universal vaccines employing heterologous modalities with improved mucosal responses, durability and breadth are vital for protection against future evolving respiratory virus strains. The intranasal influenza viral vector SARS-CoV-2 M2SR has the potential to provide improved protection against the infection and transmission of influenza and SARS-CoV-2 variants.

## Data Availability

The data presented in this study are available on request from the corresponding author.

## Supplementary Materials

The following supporting information can be downloaded at: https://www.mdpi.com/article/10.3390/vaccines11061063/s1, Table S1: Intranasal COVID Vaccines in Clinical Development.
