# Supplementary material for: Intranasal Single-Replication Influenza Vector Induces Cross-Reactive Serum and Mucosal Antibodies against SARS-CoV-2 Variants
Source: Vaccines (Basel). 2023 Jun 5;11(6):1063. doi: 10.3390/vaccines11061063 (PMC10302585; doi:10.3390/vaccines11061063)
Supplement: Supplementary file 1 [file vaccines-11-01063-s001.zip › vaccines-2408192-supplementary.pdf]

Supplementary Table S1: Intranasal COVID Vaccines in Clinical Development.

**Developer**	**Vaccine**	**Status**
Altimmune	AdCOVID, adenovirus vector	Abandoned
Bharat Biotech	BBV154, Chimp adenovirus encoding spike SARS-CoV-2 spike	Phase 3
Cansino	Convidecia Air™, adenovirus Type5 encoding SARS-CoV-2 spike for inhalation	Approved in China
AstraZeneca	ChAdOx1-S, chimpanzee adenovirus encoding the SARS-CoV-2 spike protein	Phase 1 complete
Gamaleya	Sputnik V vaccine, adenoviruses encoding SARS-CoV-2 spike	Approved in Russia
Codagenix	CoviLiv, attenuated SARS-CoV-2	Phase 2/3
Meissa Vaccines	MV-014-212, attenuated RSV vector encoding SARS-CoV-2 spike	Phase 1
Cyan Vac	CVXGS1, SARS-CoV-2 spike in canine parainfluenza virus	Phase 1
University of Hong Kong, Xiamen University	DelNS1-2019-nCoV-RBD-OPT1, attenuated influenza virus encoding SARS-Cov-2 spike	Phase 3
Icahn School of Medicine at Mount Sinai	NDV-HXP-S, Newcastle disease virus displaying SARS-CoV-2 spike	Phase 2/3
Tetherex Pharmaceuticals	SC-Ad6-1, human adenovirus encoding SARS-CoV-2 spike	Phase 1
